# Prevalence, sociodemographic factors and psychological distress related to compulsive buying online

**DOI:** 10.1192/j.eurpsy.2024.837

**Published:** 2024-08-27

**Authors:** M. Mnif, F. Guermazi, D. Mnif, W. Abid, I. Feki, I. Baati, J. Masmoudi

**Affiliations:** CHU Hedi Chakeur psychiatry A department, Sfax, Tunisia

## Abstract

**Introduction:**

Since that the online commerce provides an important shopping environment, it has been argued that traditional buying-shopping disorder may migrate into the online market.

**Objectives:**

The aims of the current study were to investigate the prevalence of online buying-shopping disorder, and to determine sociodemographic and psychological factors related to this addictive behavior.

**Methods:**

A cross-sectional, descriptive and analytical study was conducted among subjects who had already made at least one online shopping. Data was collected using a self-questionnaire published by GOOGLE FORMS. Assessment included the short version of the Internet Addiction Test modified for online shopping sites (s-IATshop). The Hospital Anxiety and Depression Scale (HADS) has been used to assess anxiety and depression.

**Results:**

A total of 137 participants aged 34.62 ± 9.82 years completed the online questionnaire.

Only 4 (2.9%) participants had a probable compulsive buying shopping on line.

The HADS-A score ranged from 0 to 14, with a mean of 6.85 +/- 3.49 and almost half of the participants (44.5%; N=61) had anxiety symptoms.

A high s-IAT shopping score was correlated with secondary or university education (p=0.046). We also found that women, who were younger and had higher incomes, had the highest scores on the s-IAT-shopping scale, without however confirming statistical significance.

**Conclusions:**

Our study has shown the potential vulnerability factors for compulsive online shopping disorder. Thus, this behaviour deserves to be taken into account in behavioural addictions.

**Disclosure of Interest:**

None Declared

